# Mitochondrial dysfunction in Alzheimer's disease and related sex differences

**DOI:** 10.3389/fnagi.2026.1761702

**Published:** 2026-03-24

**Authors:** Afzal Misrani, Conelius Ngwa, Fudong Liu

**Affiliations:** Department of Neurology, McGovern Medical School, The University of Texas Health Science Center at Houston, Houston, TX, United States

**Keywords:** Alzheimer's disease, dementia, mitochondria, oxidative stress, sex differences

## Abstract

Alzheimer's disease (AD), the most common form of dementia, accounts for 70% of cases and remains a major healthcare challenge due to its rising prevalence and lack of disease-modifying treatments. Clinically, AD is a sexually dimorphic disease. Women exhibit more rapid cognitive decline and accelerated brain atrophy during mild cognitive impairment and early dementia, whereas men more frequently present cardiovascular comorbidities, earlier mitochondrial dysfunction, and greater neuropsychiatric symptoms. AD is marked by amyloid-β (Aβ) plaques, neurofibrillary tangles, neuroinflammation, and neuronal loss, with mitochondrial dysfunction emerging as a key early contributor that exhibits sex specific phenotypes. Mitochondria are vital for neuronal function by generating ATP, maintaining calcium homeostasis, and regulating oxidative stress. However, mitochondria in AD exhibit impaired ATP synthesis, excessive reactive oxygen species (ROS) production, calcium dysregulation, and disrupted fission-fusion dynamics. AD mitochondrial dysfunction can be measured by molecular markers, such as increased expression of fission-related protein Drp1, decreased biogenesis regulator PGC-1α, and elevated oxidative stress markers like malonaldehyde, nitotyrosine and protein carbonyls. Accumulating data suggest that sex differences in mitochondrial dysfunction are attributed to either sex hormonal or sex chromosomal effects, which eventually contribute to sex dichotomic phenotypes of AD. This review collected data regarding mitochondrial dysfunction in AD, with an emphasis on sex differences in oxidative stress, energy metabolism, and regulatory pathways.

## 1 Introduction

AD is the leading cause of dementia, accounting for approximately 70% of cases, and poses a significant healthcare challenge due to its substantial caregiving burden. Neuropathologically, AD is characterized by extracellular accumulation of misfolded and aggregated Aβ peptides, along with intraneuronal neurofibrillary tangles composed of hyperphosphorylated tau (p-tau) protein. Advanced age and female sex are among the most prominent risk factors (Ferretti et al., 2018). The disease manifests as progressive neuronal degeneration, particularly affecting the cortex and hippocampus, with mitochondrial dysfunction and neuroinflammation implicated as early contributors to its pathogenesis (Wang et al., 2020; Misrani et al., 2021b; Song et al., 2021). Due to the lack of effective disease-modifying treatments, the global prevalence of AD is projected to exceed 50 million by 2050. The complexity and heterogeneity of AD, compounded by repeated failures of potential therapies in clinical trials, underscore the urgent need for deeper mechanistic insights to facilitate the development of efficacious therapeutic strategies (Gauthier et al., 2016).

Sex differences have long been found in AD epidemiology and pathology. While women are disproportionately affected due to greater longevity, men with AD experience more rapid cognitive decline and higher mortality rates across demographic groups, suggesting a male disadvantage in both early- and late-onset cases (Beam et al., 2018; Zhu et al., 2021; Emrani and Sundermann, 2025). Cognitive and psychiatric symptoms also differ between sexes, with women showing cognitive decline following a diagnosis of mild cognitive impairment (MCI) or AD dementia (Sohn et al., 2018). Additionally, brain atrophy rates and patterns vary along the AD continuum, with women exhibiting faster atrophy than men during MCI (Elbejjani et al., 2015). Men with AD, however, often exhibit more pronounced cardiovascular comorbidities (Santiago and Potashkin, 2021; Nowell et al., 2025), earlier mitochondrial dysfunction, and greater agitation and aggression, all of which may exacerbate disease progression and contribute to higher mortality (Brodaty et al., 2015). Earlier mitochondrial dysfunction can be clinically detected by reduced cerebral glucose metabolism on Fluorodeoxyglucose-Positron Emission Tomography (FDG-PET), often preceding significant structural changes on Magnetic resonance imaging (MRI; Tenney et al., 2014). It may also be reflected by increased oxidative stress biomarkers, reduced glutathione levels, impaired adenosine triphosphate (ATP) production, and mitochondrial DNA damage in blood or cerebrospinal fluid (CSF). Nevertheless, the mechanisms underlying these sex-specific differences in AD remain poorly understood. Sex differences also exist in therapeutic responses in AD. For instance, women with AD treated with donepezil exhibit lower mortality rates than men (Haywood and Mukaetova-Ladinska, 2006) and show greater clinical responsiveness to both donepezil and rivastigmine (Scacchi et al., 2014). These sex-related variations in clinical presentation, behavioral profiles, and region-specific neuropathology may necessitate more tailored management strategies. However, until relatively recently, clinical trial recruitment and study design have largely ignored the potential impact of sex on therapeutic outcomes, and results were frequently reported without sex-stratified analyses. Although contemporary AD trials more closely reflect the higher disease prevalence in women, systematic reporting of sex-specific drug responses remains inconsistent and warrants further attention.

Mitochondria are the principal organelles responsible for cellular bioenergetics, generating ATP, a crucial coenzyme required for neuronal viability and optimal function (Spinelli and Haigis, 2018). Emerging evidence indicates a reduction in mitochondrial ATP levels within the affected brain regions of both AD patients and corresponding mouse models (Gauba et al., 2019; Wang et al., 2020; Misrani et al., 2021b). Mitochondrial dysfunction is associated with an upregulation of ROS production, which exacerbates oxidative stress, promotes neuroinflammation, and contributes to progressive neuronal degeneration in AD. It has been suggested that reduction in estrogen levels during aging is associated with heightened oxidative stress and mitochondrial dysfunction (Beckman and Ames, 1998). There has been limited research on sex differences in mitochondrial dysfunction in AD, despite accumulating evidence of sex-specific differences in its role in AD progression. Understanding these differences could provide crucial insights for developing more effective, sex-specific therapeutic strategies for AD. This review discusses recent advances in altered mitochondrial pathways in AD that exhibit sex differences.

## 2 Mitochondrial dysfunction in AD neurons

Mitochondria are indispensable for neuronal function, serving as the principal source of ATP while orchestrating calcium homeostasis, synaptic plasticity, and neuronal development and survival. They are critical for axonal outgrowth, maintenance of redox equilibrium, and the regulation of neuronal excitability and intercellular communication within the brain (Saxton and Hollenbeck, 2012; Lopez-Domenech and Kittler, 2023). Mitochondria in glial cells provide essential metabolic substrates to neurons, while mitochondrial dynamics, including fusion and fission, regulate bioenergetic homeostasis. Mitochondrial fission and fusion are dynamic processes that regulate mitochondrial morphology and function, ensuring energy demands are met and damaged mitochondria are removed (Liu et al., 2020; Adebayo et al., 2021). Fission splits mitochondria, while fusion combines them, and both processes are essential for maintaining mitochondrial health and function. The balance between fission and fusion is essential for maintaining healthy mitochondria and proper cellular function. Disruptions in this balance can lead to mitochondrial dysfunction and contribute to the development of AD (Wang et al., 2009b; Zhu et al., 2013). In neural stem and progenitor cells, mitochondrial morphology and function influence cell fate decisions (Ma et al., 2020). Despite constituting only ~2% of total body mass, the human brain accounts for ~20% of the body's total metabolic expenditure (Attwell and Laughlin, 2001), consuming up to 25% of systemic glucose, primarily metabolized through glycolysis and oxidative phosphorylation (OXPHOS) to sustain synaptic activity (Yin et al., 2016). Notably, neurons utilize 70–80% of this energy demand, with the remaining fraction allocated to glial cells, including astrocytes, oligodendrocytes, and microglia (Camandola and Mattson, 2017). Due to their high metabolic rate and limited regenerative capacity, neurons are particularly vulnerable to mitochondrial dysfunction. Mitochondrial dysfunction in AD does not affect all neuronal populations uniformly; instead, it arises in a subtype-specific manner. Excitatory neurons exhibit the earliest and most substantial mitochondrial impairments, including reduced oxidative phosphorylation, decreased ATP generation, and elevated production of ROS (Wang et al., 2009a; Sun et al., 2013; Cai and Tammineni, 2017). These cells are intrinsically energy-demanding, requiring sustained synaptic activity and long-range signaling to support cognitive function. As a result, even modest disruptions in mitochondrial function may place excitatory neurons at disproportionate risk. Notably, mitochondrial dysfunction in excitatory neurons closely parallels core pathological features of AD. Impaired mitochondrial metabolism is strongly associated with amyloid-β toxicity, tau pathology, synaptic dysfunction, and neuronal loss (Reddy, 2011; Torres et al., 2021). Consistent with these observations, single-cell and spatial transcriptomic analyses of AD brains repeatedly reveal downregulation of mitochondrial and bioenergetic gene programs within excitatory neuron populations (Zou et al., 2024). Together, these findings support the idea that mitochondrial failure is not merely a downstream consequence of neurodegeneration, but may actively contribute to the selective vulnerability of excitatory neurons. In contrast, inhibitory neurons appear relatively more resilient, particularly during early stages of disease (Castanho et al., 2025). Mitochondrial alterations in these cells are often subtler or delayed and are less frequently associated with overt neuronal loss. However, mitochondrial dysfunction in inhibitory neurons may still have important functional consequences (Guo et al., 2017; Zhang et al., 2020). Rather than driving cell death, such changes may contribute to neural circuit instability and disruption of excitatory–inhibitory balance, potentially exacerbating network dysfunction and cognitive impairment. Importantly, vulnerability may vary among interneuron subtypes, indicating that the resistance is relative rather than absolute.

In AD, impaired cholinergic neurotransmission is a central contributor to deficits in memory and learning (Hampel et al., 2019; Chen Z. R. et al., 2022). This decline results from multiple converging mechanisms, including loss of cholinergic neurons in the basal forebrain, hippocampus, and amygdala, disrupted acetylcholine metabolism, and downregulation of nicotinic receptors, except for the α7 subtype. Together, these alterations lead to a sustained reduction in cholinergic signaling and cognitive dysfunction. Emerging evidence suggests that mitochondrial dysfunction plays a critical role in driving these cholinergic deficits (Wong et al., 2020). Cholinergic neuronal loss is closely associated with excessive permeabilization of the mitochondrial membrane, opening of the mitochondrial permeability transition pore (mPTP), and subsequent release of pro-apoptotic factors such as cytochrome c. This cascade activates caspase-9 and caspase-3, ultimately triggering apoptotic cell death. Increased mitochondrial membrane permeability therefore represents a key mechanistic link between mitochondrial dysfunction and cholinergic neurodegeneration. The resulting loss of cholinergic neurons exacerbates ACh insufficiency, reinforcing synaptic failure and accelerating cognitive decline in AD.

Neuronal information processing is an energetically demanding process, requiring substantial ATP consumption. It has been estimated that a single resting cortical neuron utilizes over 4.7 × 10^9^ ATP molecules per second (Zhu et al., 2012), and this demand increases significantly during action potential generation. Additional energetic costs arise from intracellular transport of biomolecules across the extensive axonal and dendritic networks, as well as from protein synthesis in response to synaptic activity. Cellular energy metabolism is governed by the interplay between cytosolic glycolysis and mitochondrial OXPHOS. Glucose serves as the primary energy substrate for the brain, first undergoing glycolysis in the cytoplasm of neurons or astrocytes, where it is converted to pyruvate. Pyruvate is subsequently transported into mitochondria and decarboxylated to generate acetyl-coenzyme A (acetyl-CoA). Fatty acid β-oxidation, occurring in both mitochondria and peroxisomes, provides an additional source of acetyl-CoA. Furthermore, ketone bodies, produced from hepatic fatty acid oxidation and released into the circulation, serve as an alternative energy substrate for extrahepatic tissues, including the brain (Kolb et al., 2021; Puchalska and Crawford, 2021). Acetyl-CoA derived from these metabolic pathways enters the tricarboxylic acid (TCA) cycle, generating the NADH and FADH_2_, which subsequently feed into the electron transport chain to drive ATP synthesis. Dysregulation of these processes can result in cellular stress and damage, contributing to pathological conditions. Mitochondrial dysfunction is a key factor in aging and the pathogenesis of neurodegenerative disorders such as AD (Misrani et al., 2021b; Zhang et al., 2025). Impairments in mitochondrial dynamics, including fission, fusion, and intracellular transport, disrupt neuronal integrity, leading to synaptic failure and neurodegeneration.

### 2.1 Mitochondrial dysfunction in microglia in AD

Microglia are the resident immune cells in the CNS. They are involved in immune surveillance, modulating neuroinflammation, clearance of debris and neuronal repair (Patel et al., 2013). In AD, the function of microglia becomes highly context dependent. When maintained at physiologic levels, they are protective by clearing amyloid-β and maintaining tissue homeostasis. Upon chronic stimulation by pathologic insults, microglia become persistently activated and release pro-inflammatory molecules like tumor necrosis factor-α and interleukin-1β, promoting neuroinflammation and neuronal damage (Leng and Edison, 2021; Valiukas et al., 2025). Growing evidence indicates that metabolic disturbances (particularly mitochondrial dysfunction) are central drivers of maladaptive microglial behavior in AD (Yang et al., 2025). Mitochondria provide the energy required for phagocytosis, migration, and immune signaling, while also regulating redox balance, calcium homeostasis, apoptosis, and polarization states. Disruptions in mitochondrial function, including impaired energy metabolism, increased oxidative stress, altered mitochondrial dynamics, defective autophagy, and dysregulated calcium handling, not only undermine neuronal health but also bias microglia toward a pro-inflammatory activation state (summarized in Table 1). These alterations diminish phagocytic capacity, enhance inflammatory signaling, and increase microglial apoptosis, ultimately reinforcing a self-sustaining cycle of microglial dysfunction that accelerates AD progression (Agrawal and Jha, 2020; Li et al., 2024).

**Table 1:** Mitochondrial dysfunction in microglia in AD.

**Mitochondrial pathway**	**Key signaling molecules**	**Mitochondrial alterations in AD microglia**	**Functional consequences in microglia and AD pathology**	**References**
Bioenergetic metabolism (OXPHOS)	ETC complexes I–IV, ATP synthase, AMPK	Reduced oxidative phosphorylation, ATP deficiency	Impaired phagocytosis and migration, reduced Aβ clearance, plaque accumulation	Sangineto et al., 2023; Wu et al., 2025
Oxidative stress regulation	mtROS, Nrf2, SOD2	Excess mitochondrial ROS production	Enhanced inflammatory signaling, neuronal oxidative damage	Colton et al., 2000; Simpson and Oliver, 2020
Mitochondrial dynamics (fission/fusion)	Drp1, Fis1, Mfn1/2, OPA1	Excessive fission and mitochondrial fragmentation	Pro-inflammatory microglial polarization, chronic neuroinflammation	Chiurazzi et al., 2020; Blagov et al., 2022
Mitophagy/quality control	PINK1, Parkin, BNIP3, LC3	Impaired clearance of damaged mitochondria	Accumulation of dysfunctional mitochondria, sustained inflammatory activation	Lautrup et al., 2019; Eshraghi et al., 2021; Lin et al., 2022
mPTP opening & apoptosis	Cyclophilin D, cytochrome c, caspase-9/3	Increased mitochondrial membrane permeabilization	Microglial apoptosis or dysfunction, loss of homeostatic support	Bamberger and Landreth, 2002; Lee et al., 2019
Calcium homeostasis	MCU, NCLX, ER–mitochondria contacts (MAMs)	Dysregulated mitochondrial Ca^2+^ handling	Aberrant cytokine release, excitotoxic neuronal injury	McLarnon et al., 2005; McLarnon, 2020
Inflammasome activation	NLRP3, ASC, caspase-1	mtROS and mtDNA-driven inflammasome activation	Increased IL-1β/IL-18 release, amplified neuroinflammation	Goldmann et al., 2013; Hanslik and Ulland, 2020
Immunometabolic polarization	NF-κB, HIF-1α, PGC-1α	Suppressed mitochondrial biogenesis and oxidative metabolism	Shift toward pro-inflammatory phenotypes, reduced neuroprotection	Fairley et al., 2021; Singh and Singh, 2025
Mitochondrial biogenesis	PGC-1α, NRF1/2, TFAM	Impaired mitochondrial renewal	Reduced metabolic flexibility, vulnerability to chronic stress	Kang et al., 2018; Mi et al., 2021
Innate immune signaling	mtDNA, cGAS–STING, TBK1	Cytosolic mtDNA release	Type I interferon responses, sustained innate immune activation	Govindarajulu et al., 2023; Li et al., 2025

Whether microglia exhibit sex-dependent differences under homeostatic conditions has been the subject of extensive investigation, with growing evidence indicating that these differences vary with age and brain region. During the first postnatal week, male mice show a greater abundance of phagocytic, amoeboid microglia in the amygdala, whereas females display higher numbers in the hippocampus (Nelson et al., 2017; VanRyzin et al., 2020). This divergence continues into later developmental stages, with males demonstrating increased microglial density in both the hippocampus and cortex (Guneykaya et al., 2018). These findings support the notion that male and female microglia adopt distinct phenotypic profiles.

Mechanistically, sex-specific differences in microglial gene expression and functional responses appear to arise from the combined, context-dependent influences of sex chromosomes and sex hormones, which may interact synergistically or antagonistically across molecular pathways (Palaszynski et al., 2005). Advances in single-cell and single-nucleus sequencing have further refined our understanding of microglial heterogeneity in AD across human and experimental animal tissues. Notably, female mice exhibit sex-dependent microglial activation in response to amyloid pathology, but not tau pathology, indicating pathway-specific distinctions in microglial reactivity (Biechele et al., 2020). Collectively, these observations highlight sex as a critical biological variable shaping microglial activation, with important implications in neuroinflammatory processes in AD.

### 2.2 Mitochondrial dysfunction in astrocytes in AD

Astrocytes play essential roles in supporting neuronal function by supplying metabolic substrates, regulating synaptic plasticity, and modulating neuronal activity (Pessoa et al., 2026). A central component of this support involves maintaining neurotransmitter homeostasis. Astrocytes buffer excess glutamate by converting it to glutamine through glutamine synthetase and mitochondrial tricarboxylic acid cycle activity, thereby preserving synaptic integrity and preventing excitotoxicity (Hertz and Rothman, 2017; Fernandez-Gonzalez and Galea, 2023). Compared with neurons, astrocytes exhibit a predominantly glycolytic metabolic profile, enabling them to sustain high energetic demands and provide metabolic support to surrounding cells (Bonvento and Bolanos, 2021). Disruption of astrocytic metabolism has been increasingly implicated in AD pathogenesis. Pharmacological inhibition of glycolytic enzymes in astrocytes promotes Aβ accumulation in the brain, indicating that astrocytic glycolysis contributes to amyloid homeostasis (Allaman et al., 2010; Fu et al., 2015). In addition, ~20% of cerebral energy supply is derived from fatty acid oxidation, which occurs predominantly in astrocytes (Belanger et al., 2011), further highlighting their central role in brain energy metabolism. Transcriptomic analyses of astrocytes from AD brains reveal significant alterations in mitochondrial and immune-related genes, indicating heightened vulnerability to disease-associated stress. Compared with controls, 226 genes were differentially expressed, with 55.8% upregulated, including mitochondrial genes Fas-activated serine/threonine phosphoprotein kinase domains 2 (FASTKD2), tRNA methyltransferase 61 homolog B (TRMT61B), pitrilysin metalloproteinase 1 antisense RNA 1 (PITRM1-AS1) and immune-related genes clusterin, apolipoprotein J (CLU), complement component 3 (C3), cluster of differentiation 74 molecule (CD74; Sekar et al., 2015). Consistent with these molecular changes, increased oxidative stress has been observed in astrocytes from hAPP mouse models, indicating that redox imbalance contributes to astrocytic dysfunction (Lee et al., 2012). Additionally, exposure of Aβ to astrocyte can induce mitochondrial fragmentation and depolarization, therefore leading to increased ROS production and metabolic impairment (Sarkar et al., 2014). Moreover, transcriptomic profiling of human astrocytes confirms downregulation of nuclear-encoded genes involved in the tricarboxylic acid cycle and electron transport chain, pointing to defects in mitochondrial energy production (Galea et al., 2022). Mitochondrial biogenesis represents a critical mechanism regulating astrocyte maturation and synaptic support and is partly mediated by the metabolic regulator peroxisome proliferator-activated receptor-γ coactivator 1-α (PGC-1α; Zehnder et al., 2021). *In vivo* studies demonstrate that astrocyte-specific PGC-1α deficiency disrupts neuronal synapse formation and function, emphasizing the importance of mitochondrial renewal in maintaining neural connectivity. Accordingly, restoring astrocytic mitochondrial biogenesis may represent a promising therapeutic strategy for AD. Recent evidence also highlights sex-specific differences in astrocytic mitochondrial function. Astrocytes derived from female AD patients exhibit increased mitochondrial membrane potential, elevated hydrogen peroxide levels, and enhanced superoxide production compared with male-derived cells (Flannagan et al., 2023). These findings suggest that sex-dependent mitochondrial vulnerability in astrocytes may also contribute to AD progression and therapeutic responses. Collectively, impaired mitochondrial function in astrocytes compromises their metabolic and neuroprotective roles, promotes neuroinflammation, and establishes a feed-forward cycle that exacerbates neuronal injury in AD.

## 3 Mechanisms of mitochondrial dysfunction in Alzheimer's disease: oxidative stress, energy failure, calcium dysregulation, and mitochondrial dynamics

### 3.1 Oxidative stress

Metabolic alterations are detectable in the early stage of AD, with impaired energy metabolism preceding cognitive decline (Lopez-Lee et al., 2024). Notably, sex differences in mitochondrial dysfunction in AD have been increasingly reported (Ferretti et al., 2018; Jimenez-Herrera et al., 2024). Positron emission tomography (PET) imaging reveals abnormally reduced glucose metabolism in multiple brain regions of AD patients, which correlates with the disease severity (Alexander et al., 2002). Women initially undergo a degree of protection against mitochondrial dysfunction, as evidenced by cognitive advantages in mild-to-moderate AD compared to men. However, these advantages diminish when adjusted for metabolic rate or as disease pathology progresses (Sundermann et al., 2020). Emerging evidence suggests that female cells exhibit greater resistance to oxidative damage compared to male cells (Matarrese et al., 2011). Additionally, female animals demonstrate lower ROS levels and reduced mitochondrial DNA damage relative to their male counterparts (Borras et al., 2003; Vina et al., 2003). Intriguingly, sex-related differences in oxidative stress have been observed in individuals with AD (Scheff et al., 2016). For example, Homocysteine levels increased with age in both men and women with AD, with men exhibiting significantly higher levels, indicating greater oxidative stress in male vs. female patients (Tenkorang et al., 2018). Moreover, sex differences in oxidative stress in AD persist (Kayali et al., 2007; Dugan et al., 2009). Additionally, glutathione levels are lower in the spleen and brain cells of male mice, indicating a higher oxidative status in males compared to females (Viveros et al., 2007). Consistently, male AD patients exhibit lower glutathione concentrations in red blood cells than female AD patients and healthy age-matched controls. The authors proposed that this reduction in glutathione, a primary cellular antioxidant, may increase susceptibility to AD in men (Beam et al., 2018).

The precise role of oxidative damage in the onset and early progression of AD remains unclear. However, oxidative stress, characterized by an imbalance between ROS production and antioxidant defense mechanisms, is recognized as an early event in AD pathogenesis (Arimon et al., 2015; Scheff et al., 2016; Misrani et al., 2021b). Oxidative stress and Aβ are closely interconnected; Aβ promotes the generation of oxidative stress, while oxidative stress, in turn, enhances Aβ accumulation. This self-perpetuating cycle makes it difficult to determine which process initiates the pathology (Arimon et al., 2015; Tamagno et al., 2021). However, compelling evidence indicates that oxidative stress occurs early in AD and progressively accumulates, ultimately worsening key pathological features such as Aβ plaque formation, neurofibrillary tangles, metabolic dysfunction, and cognitive decline. Notably, ROS-driven Aβ accumulation leads to lysosomal membrane degradation, ultimately promoting neuronal death (Zhang et al., 2009). Oxidative imbalance and a marked increase in its by-products, such as 4-hydroxynonenal (4-HNE), malondialdehyde (MDA), 3-nitrotyrosine and protein carbonyls, have been consistently reported in AD. A significant body of research highlights an upregulation in lipid peroxidation, a process in which ROS attack lipids, generating peroxidation products through a free radical chain reaction (Galbusera et al., 2004). Elevated levels of MDA, a major by-product of lipid peroxidation, have been detected in the hippocampus, pyriform cortex (Lovell et al., 1995) and erythrocytes of AD patients (Dei et al., 2002). Given its ease and cost-effectiveness, MDA measurement may serve as a valuable biomarker for monitoring AD progression and evaluating treatment efficacy. In humans, plasma MDA level has been found higher in men vs. women in both young and aging population (Ide et al., 2002; Beam et al., 2018). Overall, these findings indicate that sex differences exist in mitochondrial oxidative stress, which may contribute to the sexually dimorphic phenotypes of AD.

### 3.2 Mitochondrial biogenesis

Mitochondrial biogenesis is the process by which mitochondria expand in both number and size, ensuring the continuous renewal necessary for maintaining a functional mitochondrial network. This process is primarily regulated by peroxisome proliferator-activated receptor gamma coactivator-1 (PGC-1), a transcriptional coactivator that orchestrates the expression of nuclear-encoded mitochondrial genes essential for organelle function. PGC-1α, a key isoform, interacts with various transcription factors, including nuclear respiratory factor (NRF)-1, NRF-2, peroxisome proliferator-activated receptors (PPARα, PPARδ, PPARγ), and estrogen-related receptor α, to modulate mitochondrial gene expression (Lin et al., 2005). In the brain, disruption of PGC-1α activity induces mitochondrial dysfunction, leading to neuronal degeneration (St-Pierre et al., 2006). Research indicates that elevated PGC-1α levels confer neuroprotection by upregulating antioxidant gene expression, thereby mitigating oxidative stress-induced apoptosis in neural cells (St-Pierre et al., 2006). Additionally, PGC-1α plays a crucial role in regulating mitochondrial density in neurons (Wareski et al., 2009), as evidenced by increased susceptibility to neuronal degeneration in PGC-1α-knockout mice (McMeekin et al., 2018; Tang et al., 2024). Suppression of PGC-1α is associated with reduced oxygen consumption and glucose oxidation, as well as increased expression of OXPHOS proteins, ultimately resulting in elevated ROS production (Sahin et al., 2011). PGC-1α downregulation has been reported to be associated with altered mitochondrial dynamics, increased ROS production, Ca^2+^ dyshomeostasis, reduced oxidative phosphorylation, and disrupted ATP production (Dabrowska et al., 2015; Peng et al., 2017; Panes et al., 2022). Growing evidence suggests that PGC-1α pathway impairment contributes to early synaptic loss in AD-affected regions like the hippocampus and cortex (Dong et al., 2020; Singulani et al., 2020). Its downregulation is linked to early disease onset and disrupted neuronal circuitry. Notably, PGC-1α levels decline in Tg2576 and APP/PS1 AD mice, correlating with mitochondrial dysfunction and reduced non-amyloidogenic APP processing (Pedros et al., 2014; Dabrowska et al., 2015). The capacity for mitochondrial biogenesis declines with aging and in neurodegenerative disorders. Reduced PGC-1α levels have been reported in both AD mouse models (Singulani et al., 2020; Wang et al., 2022) and patients (Qin et al., 2009), whereas PGC-1α overexpression in 2xTg-AD or APP/PS1 mice has been shown to reduce Aβ plaque burden, restore mitochondrial fission-fusion balance, and mitigate oxidative damage (Wang et al., 2021). Furthermore, treatment with resveratrol (RSV), a SIRT1/PGC-1α pathway activator, improved cognitive function, learning ability, and spatial memory in Aβ1-42 rats and APP/PS1 transgenic mice (Wang R. et al., 2017). Overall, these findings suggest that PGC-1α plays a neuroprotective role in mitigating neuronal loss and synaptic dysfunction in AD. By regulating mitochondrial dynamics, reducing oxidative stress, and enhancing synaptic maintenance, PGC-1α may serve as a potential therapeutic target for preserving cognitive function and slowing disease progression.

**Figure 1:**
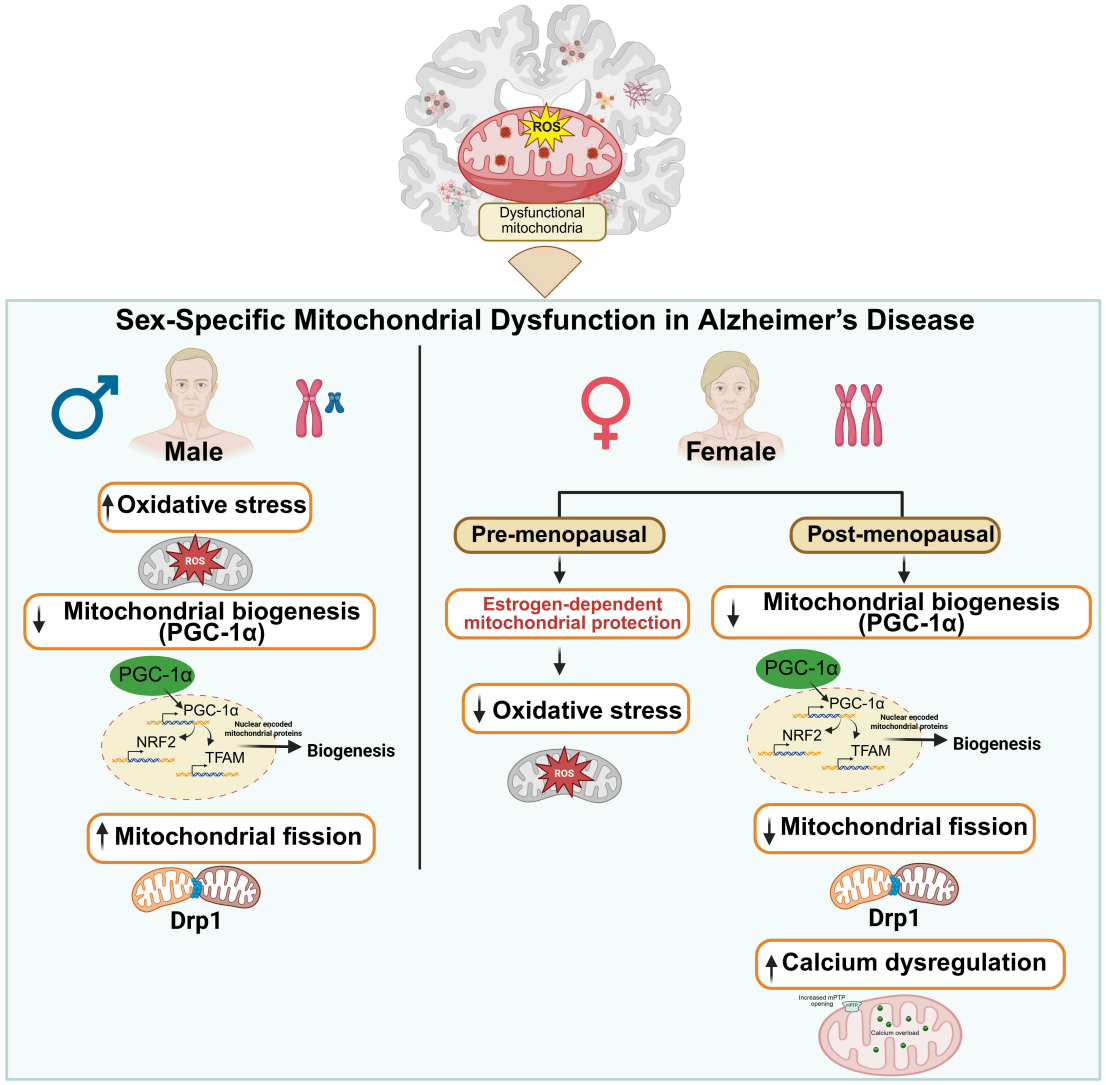
Sex differences in mitochondrial dysfunction in AD. Males exhibit higher levels of oxidative stress compared to females, which contributes to reduced mitochondrial biogenesis, primarily via decreased PGC-1α activity that leads to increased Drp1 signaling; in turn, mitochondrial fission is increased. In contrast, premenopausal females benefit from estrogen-mediated protection that supports mitochondrial function and limits oxidative damage. However, this advantage diminishes with age. Although postmenopausal females also show reduced mitochondrial biogenesis (lower PGC-1α), they display heightened intracellular calcium levels compared to age-matched males. As a result, reduced mitochondrial fission is seen in aged females. The figure was created with Biorender.com.

Potential sex differences in PGC-1α expression and function in AD have been reported in literature, although the driving force (gonadal hormones vs. sex chromosomes) is not clear. Estrogens exert a beneficial impact on mitochondrial function through multiple mechanisms, including the enhancement of mitochondrial biogenesis, attenuation of oxidative stress, and inhibition of mitochondrial cell death. Estrogen receptors are localized within mitochondrial matrix and inner mitochondrial membrane (IMM), where they regulate the expression of mitochondrial genes such as NADH, cytochrome C, ATP synthase, PGC-1α, TFAM, NRF1, NRF2, etc. (Chen et al., 2004; Chen and Yager, 2004; Yang et al., 2004; Mattingly et al., 2008; Ventura-Clapier et al., 2019). Additionally, estrogens modulate the expression of various regulatory elements, including microRNAs (miRNAs) and long non-coding RNAs (lncRNAs), that govern mitochondrial biogenesis, potentially contributing to the observed female advantage in early life (Sedano et al., 2020; Burgos et al., 2021; Colella et al., 2021). Estrogens directly upregulate the expression of nuclear-encoded mitochondrial genes, including the master regulator of energy metabolism and mitochondrial biogenesis, PGC-1α, along with its downstream targets (Hsieh et al., 2005; Mattingly et al., 2008). Notably, estrogen treatment has been shown to increase PGC-1α expression and mitochondrial protein levels in ovariectomized (OVX) mice (Chen et al., 2015). However, emerging evidence indicates that sex chromosomes contribute to sex-specific vulnerability in AD, particularly through X-linked genes that escape X-chromosome inactivation (Davis et al., 2020). While females possess two X chromosomes and males are hemizygous (one X and one Y chromosome), dosage compensation is achieved in females through X-chromosome inactivation (XCI), a developmentally regulated process that transcriptionally silences one X chromosome (Lambert et al., 2023). Notably, several X-linked genes have been associated with brain aging phenotypes (Mallard et al., 2021), supporting a potential biological contribution to female longevity and differential AD susceptibility (Davis et al., 2019; Gadek et al., 2025). Collectively, these findings underscore the emerging role of sex chromosomes as important biological determinants in AD pathogenesis. Elucidating these sex chromosome–dependent mechanisms may facilitate the development of sex-specific therapeutic strategies targeting mitochondrial dysfunction in AD (Figure 1).

### 3.3 Regulation of mitochondrial calcium homeostasis

Mitochondria play a central role in maintaining cellular Ca^2+^ homeostasis, which is essential for various neuronal functions, including neurotransmitter release, cellular motility, metabolic regulation, synaptic plasticity, proliferation, gene expression, and apoptosis. Dysregulation of Ca^2+^ signaling has been widely recognized as a key factor in accelerating AD pathology (Wang Y. et al., 2017; Garcia-Casas et al., 2023; Wang et al., 2025). Mitochondrial Ca^2+^ overload can trigger cell death by inducing mitochondrial permeability transition (PT). The PT is characterized by an increase in the permeability of the inner IMM to ions and solutes, mediated by the mitochondrial permeability transition pore (mPTP), a high-conductance, voltage-dependent channel that requires a critical Ca^2+^ load within the mitochondrial matrix for activation.

Excessive mitochondrial Ca^2+^ accumulation leads to increased ROS production, inhibition of ATP synthesis, mPTP opening, cytochrome c release, and subsequent activation of caspase-dependent apoptotic pathways. This dysregulation contributes to neurotoxicity; however, monitoring mitochondrial Ca^2+^ dynamics at the single-neuron level remains a significant technical challenge. Emerging research suggests notable sex differences in Ca^2+^ regulation within the AD brain, with females exhibiting greater disruptions in calcium homeostasis than males (Ibarreta et al., 1997; Sepulveda-Falla et al., 2014). These differences may be influenced by sex hormones or sex chromosomes and could involve distinct calcium signaling pathways in various brain cell types, including neurons (Nilsen and Diaz Brinton, 2003; Sarkar et al., 2008; Arimon et al., 2015), microglia (Wu et al., 2016) and astrocytes (Herraiz-Martinez et al., 2022). The exacerbated disruption of calcium homeostasis in females can trigger cell death and may contribute to the increased prevalence of AD in women, highlighting the need for further exploration of sex-specific mechanisms governing Ca^2+^ dysregulation and their therapeutic implications. Estrogen, the predominant female sex hormone, has been implicated in the regulation of calcium homeostasis, exerting neuroprotective effects that may mitigate Ca^2+^ imbalance (Ba et al., 2004; Brewer et al., 2006). However, the decline in estrogen levels during menopause exacerbates calcium dysregulation, increasing susceptibility to neurodegeneration in women.

In conclusion, mitochondrial Ca^2+^ dysregulation emerges as a critical contributor to AD pathogenesis, serving as a key driver of neuronal dysfunction and cell death. The pronounced sex differences observed in calcium homeostasis further highlight the complexity of AD, with females exhibiting a greater predisposition to Ca^2+^ dysregulation, influenced by hormonal fluctuations and/or sex chromosomal affect (particularly by X-linked genes that escape the process of X-chromosome inactivation; Davis et al., 2020). This disruption may amplify mitochondrial dysfunction, promoting oxidative stress, impairing ATP synthesis, and triggering apoptotic pathways.

### 3.4 Mitochondrial dynamics: the role of Drp1

Mitochondria are highly dynamic organelles that continuously undergo morphological and positional changes inside the cell. These changes are facilitated by processes collectively termed mitochondrial dynamics, which encompass two tightly regulated and opposing mechanisms: fission and fusion (a process where two mitochondria merge, forming a larger, interconnected network). Mitochondrial fission involves the division of a single mitochondrion into two, ensuring the separation of both the outer mitochondrial membrane (OMM) and IMM without compromising matrix protein integrity. This process is primarily mediated by the GTPase dynamin-related protein 1 (Drp1), which resides in the cytosol and translocate to the OMM upon activation. There, Drp1 interacts with key adaptor proteins, including mitochondrial fission protein 1 (FIS1), mitochondrial dynamics proteins 49 and 51 kDa (MiD49 and MiD51), and mitochondrial fission factor (MFF), to drive membrane constriction and division (Smirnova et al., 1998; Mozdy et al., 2000). Drp1 is a multifunctional protein involved in mitochondrial division, distribution, peroxisomal fragmentation, phosphorylation, SUMOylation, and ubiquitination. It plays a critical role in mitochondrial division and is essential for the proper distribution of mitochondria within axons, dendrites, and synapses. Drp1 regulates mitochondrial morphology and distribution (Sesaki et al., 2014). However, disruption of the Drp1 gene has been shown to cause the collapse of the mitochondrial membrane on one side of the cell (Otsuga et al., 1998). Drp1 overexpression leads to excessive mitochondrial fragmentation, resulting in the accumulation of defective mitochondria in neurons. In contrast, Drp1 reduction promotes mitochondrial fusion and enhances mitochondrial connectivity (Labrousse et al., 1999; Smirnova et al., 2001). Functional mutations in Drp1 result in impaired mitochondrial fission, leading to an elongated, hyper-fused mitochondrial network with defective bioenergetic and cellular functions (Frank et al., 2001). More importantly, the interaction between the mitochondrial fission protein Drp1 and Aβ induces S-nitrosylation of Drp1, which activates its GTPase activity and leads to excessive mitochondrial fragmentation. This disruption of mitochondrial dynamics contributes to synaptic dysfunction and may exacerbate neuronal damage and cognitive decline in AD (Manczak et al., 2011; Manczak and Reddy, 2012). Another study suggests that Drp1 interacts with Aβ monomers and oligomers in brains of AD patients, with these aberrant interactions becoming more pronounced as the disease progresses (Manczak et al., 2011). Using AD postmortem brains and cortical tissues from APP, APP/PS1 and 3XTg.AD mice, it was also found that Drp1 interacts with phosphorylated tau (Manczak and Reddy, 2012). Numerous studies also implicate Drp1 in apoptotic pathways, highlighting mitochondrial fission as a key regulator of programmed cell death. For instance, inhibition of Drp1 translocation through the polypeptide PPD1 delays apoptosis (Cereghetti et al., 2010), while pharmacological ablation of Drp1 prevents senecionine-induced apoptosis in hepatocytes (Yang et al., 2017). Consistent with these findings, our previous research demonstrated an increase in Drp1 expression and cleaved caspase-3 (a critical marker of apoptosis) in the prefrontal cortex and hippocampus of the APP/PS1 mouse model of AD, further suggesting an upregulated mitochondrial fission activity in the neurodegenerative disease (Misrani et al., 2021a). Maintaining the balance between mitochondrial fission and fusion is crucial for neuronal function and energy metabolism (Otera et al., 2013). Disrupting this equilibrium can have severe consequences for brain health, as evidenced by studies showing that Drp1 knockout in CA1 hippocampal neurons leads to synaptic dysfunction, hippocampal atrophy, and cognitive deficits in mice (Shields et al., 2015). Although the role of Drp1 in mitochondrial dynamics and neurodegeneration has been established, these studies have largely overlooked potential sex differences in the signaling, given the evidence suggesting that mitochondrial dynamics may be differentially regulated in males and females.

Disrupted mitochondrial dynamics in AD arises from aberrant interactions between Aβ, hyperphosphorylated tau, and Drp1 (Manczak et al., 2011; Manczak and Reddy, 2012; Baek et al., 2017). These interactions promote excessive mitochondrial fission and hinder fusion processes, ultimately impairing mitochondrial function (Manczak et al., 2011). In the frontal cortex of AD patients, there is a progressive increase in Drp1 and Fis1 as the disease advances resulting in energy deficit and neuronal damage. Studies conducted in N2a neuroblastoma cells have demonstrated that exposure to Aβ leads to a significant increase in Drp1 *in vitro* (Manczak et al., 2010). Given that most neurons are postmitotic and unable to divide through mitosis, they rely heavily on mitochondrial turnover through fission and fusion to maintain quality control and functional integrity. Defects in mitochondrial dynamics disproportionately affect neurons compared to other cell types, rendering them particularly vulnerable in neurodegenerative diseases such as AD. Interestingly, fibroblasts derived from AD patients exhibit an accumulation of elongated mitochondria in the perinuclear region, suggesting a disruption in mitochondrial fission and fusion processes (Wang et al., 2008). Several studies have reported elevated Drp1 transcript levels and increased GTPase activity in post-mortem brain samples from AD patients and murine AD models (Manczak et al., 2011; Kandimalla et al., 2018). However, conflicting results have also been observed, with some studies showing a notable reduction in Drp1 levels in the hippocampus of AD patients compared to controls (Wang et al., 2009a). Sex- and age-dependent differences in Drp1 expression have been observed in the 3xTg mouse model of AD, with male mice exhibiting higher Drp1 levels than females (Djordjevic et al., 2020). Notably, aged male AD mice show a decline in Drp1 compared to middle-aged males. Consistently, post-mortem analyses of AD patient brains have reported reduced Drp1 levels in females (Pascale et al., 2023). These discrepancies underscore the need for further investigation into sex-specific differences in Drp1 expression and function in AD. Future studies could be benefited by utilizing genetic mouse models of AD crossed with XY^*^ or four-core genotype mice (Arnold, 2020), which were designed to specifically study the effects of sex hormones or sex chromosome complement.

### 3.5 Mitophagy

Mitophagy is a selective form of autophagy critical for maintaining mitochondrial quality control by removing damaged or dysfunctional mitochondria, thereby preserving cellular homeostasis and mitigating oxidative stress. This process is regulated by key signaling pathways, including the PTEN-induced putative kinase protein 1 (PINK1)/Parkin and BNIP3/NIX pathways, and plays an essential role in neuroprotection, aging, and the pathogenesis of AD (Pradeepkiran and Reddy, 2020; Chen et al., 2021; Mary et al., 2023). In the case of cellular stress, sustained IMM depolarization leads to the stabilization of PINK1 on the OMM. PINK1 then phosphorylates Mitofusin 2 (Mfn2), which activates the ubiquitin–proteasome system (UPS) to recruit Parkin. Parkin then facilitates the encapsulation of damaged mitochondria into mitophagosomes, which are subsequently degraded by the lysosomal system. However, mitochondrial quality control mechanisms, essential for detecting and eliminating dysfunctional mitochondria, become compromised with age, AD progression, or prolonged cellular stress, leading to neuronal dysfunction, cell death, and cognitive decline (Cai and Tammineni, 2016; Baek et al., 2017; Zhang et al., 2019; Sharma C. et al., 2021). Impaired mitophagic clearance has been found to accelerate AD pathology since the early 2000s (Hirai et al., 2001). This notion is supported by the accumulation of mitochondrial DNA (mtDNA) and proteins within the cytoplasm, as well as within autophagic vacuoles (AVs) in AD neurons, which are accompanied by increased oxidative damage (Hirai et al., 2001). Evidence has shown a reduction in mitophagic degradation in the brains of AD patients, particularly in those with elevated levels of total and phosphorylated Tau (pTau) protein, suggesting that Tau may contribute to the observed impairment in this process. Furthermore, decreased mRNA and protein expression of PINK1 have been reported in the hippocampi of individuals at late-stage of AD (Braak stages V-VI; Du et al., 2017). In addition to PINK1, other key proteins involved in autophagy and mitophagy, such as Optineurin (OPTN), ATG5, ATG12, Beclin-1 (Bcl-1), PI3K class III, ULK1, AMBRA1, BNIP3, BNIP3L, FUNDC1, and VDAC1, have shown reduced expression in AD-affected brains (Martin-Maestro et al., 2017). Studies of post-mortem hippocampal brain samples from AD patients have also demonstrated a 30–50% reduction in basal mitophagic activity compared to sex- and age-matched cognitively normal controls (Fang et al., 2019). Additionally, defective mitophagy has been reported in human sporadic AD brains (Braak stages IV-VI), characterized by an increased LC3-II/I ratio and elevated levels of p62, as well as a reduction in PINK1 and Parkin levels within mitochondria-enriched fractions (Vaillant-Beuchot et al., 2021). These findings emphasize the critical role of mitophagy in mitochondrial quality control and neuronal health. Impaired mitophagic function in AD leads to the accumulation of dysfunctional mitochondria, contributing to neurodegeneration, increased oxidative stress, and neuronal dysfunction. To date, only a limited number of studies have examined sex differences in mitophagy. For instance, a recent report demonstrated higher expression of BNIP3L and BCL2L13 in 3xTg-AD female mice, whereas male mice exhibited elevated BNIP3 levels, suggesting impaired mitophagy in males (Adlimoghaddam et al., 2025). Given the accumulating evidence that mitochondrial dynamics, oxidative stress responses, and autophagy are regulated in a sex-specific manner, it is highly likely that mitophagy is also differentially regulated between males and females. The knowledge gap underscores the need for systematic investigations using both animal models and human samples to fully elucidate sex-specific mechanisms of mitophagy in neurodegenerative disease.

### 3.6 Apolipoprotein E4 (APOE4)-mediated mitochondrial dysfunction

APOE4 is the major genetic risk factor for late-onset AD (Serrano-Pozo et al., 2021), expressed in 40–65% of all AD patients, and strongly associated with Aβ accumulation and tau pathology. In addition to these hallmark features, ApoE4 directly impacts mitochondrial function, contributing to mitochondrial dysfunction and neuronal toxicity. ApoE4 expression is associated with increased mitochondrial Ca^2+^ accumulation and elevated ROS production, leading to heightened oxidative stress. Moreover, ApoE4 disrupts mitochondrial dynamics by altering the balance between fusion and fission, and impairs mitophagy-mediated quality control. Through these combined effects, ApoE4 promotes mitochondrial instability and defective mitochondrial turnover, ultimately exacerbating neuronal vulnerability and disease progression (Chen H. et al., 2022; Pires and Rego, 2023). *In vitro* studies in N2a cells showed that ApoE4 fragments (aa 1–272) interact with mitochondria and cause mitochondrial dysfunction and neurotoxicity (Chang et al., 2005). Consistent with its role in mitochondrial dysregulation, ApoE4 has been shown to impair respiratory function in both cellular and animal models. N2a cells stably expressing ApoE4 exhibit reduced levels of mitochondrial respiratory complexes I, IV, and V, while cortical neurons from NSE-ApoE4 mice display decreased expression of multiple respiratory chain subunits compared with ApoE3-expressing controls (Chen et al., 2011). Similarly, studies in ApoE4-expressing N2a cells reported diminished mitochondrial respiration and ATP production, indicating a reduced capacity to meet elevated energetic demands (Orr et al., 2019). Although the effects of ApoE4 on mitochondrial dynamics remain incompletely characterized, accumulating evidence suggests that ApoE4 disrupts mitochondrial homeostasis. In the hippocampus of ApoE4-transgenic mice, ApoE4 expression is associated with increased mitochondrial fusion and reduced fission and mitophagy (Simonovitch et al., 2019). In parallel, ApoE4 astrocytes exhibit decreased mitochondrial fission and impaired Parkin-mediated mitophagy, further compromising mitochondrial quality control (Schmukler et al., 2020). Together, these findings indicate that ApoE4 promotes defects in mitochondrial respiration and turnover, thereby contributing to cellular energetic failure and neurodegeneration in AD.

Advanced age, female sex, and the APOE4 genotype represent the three strongest risk factors for late-onset AD. While APOE4 increases disease susceptibility in both sexes, its effects on mitochondrial function in women compared with men remain incompletely understood. Epidemiological studies indicate that female APOE4 carriers develop AD earlier and at higher rates than male carriers, although both groups exhibit substantially elevated risk relative to non-carriers (Farrer et al., 1997; Jett et al., 2023). These observations highlight the presence of sex-dependent and APOE4-associated heterogeneity in disease vulnerability. The molecular mechanisms underlying this heterogeneity are an active area of research. Preclinical studies suggest that aging, sex, and APOE4 status converge to disrupt cerebral metabolism and bioenergetic homeostasis beginning in midlife, or even earlier (Riedel et al., 2016; Wang and Brinton, 2016). These early metabolic alterations underscore mitochondrial dysfunction as a central mediator linking genetic and biological risk factors to AD onset and progression.

## 4 Conclusions

This review discussed the contribution of mitochondrial dysfunction to AD and the potential sex differences that have not been well documented (Table 2). Mitochondrial dysfunction is central to AD pathology, and potentially regulated in a sexually dimorphic way. Women, who show higher AD prevalence, benefit from the neuroprotective effects of estrogen at younger ages. However, the post-menopausal reduction in estrogen levels accelerates mitochondrial dysfunction and cognitive decline; at the same time, sex chromosomal effects (particularly the X chromosome), may also impact on AD susceptibility (Bajic et al., 2019; Davis et al., 2020; Song et al., 2024). Accumulating data have demonstrated genes escaping from X chromosome inactivation (XCI) play critical roles in mediating sex differences in many diseases (Arnold et al., 2013; Qi et al., 2023), including AD (Davis et al., 2020). In AD, the XY chromosome combination is linked to greater neurodegenerative susceptibility than XX, likely reflecting the protective effects of a second X chromosome in females (Davis et al., 2020). AD is a sexually dimorphic disease, and further investigation into the mechanisms underlying the sex difference has high translational significance. Studies on AD related mitochondrial dysfunction that exhibits sex specific characteristics could lead to developing personalized therapeutic strategies for this devastating disease.

**Table 2:** Mitochondrial pathways in AD: molecular regulators, pathological alterations, functional consequences, and sex differences.

**Mitochondrial pathway**	**Key signaling molecules**	**Mitochondrial alterations in AD**	**Functional consequences in AD pathology**	**Sex differences in AD**	**References**
Mitochondrial dynamics (fission/fusion)	Drp1, Fis1, Mfn1/2, OPA1	Increased fission; reduced fusion; mitochondrial fragmentation	Reduced ATP, ↑ ROS, synaptic dysfunction	Greater fragmentation in males; hormone-dependent regulation	Djordjevic et al., 2020; Adebayo et al., 2021
Oxidative phosphorylation (OXPHOS)	Complex I–V, PGC-1α, NRF1, TFAM	Reduced ETC activity; impaired respiration	Bioenergetic failure; oxidative stress	Earlier bioenergetic decline in females; higher oxidative burden in males	Scheff et al., 2016; Tenkorang et al., 2018
Mitochondrial biogenesis	PGC-1α, SIRT1, AMPK, TFAM	Suppressed biogenesis; reduced mtDNA copy number	Reduced metabolic resilience	Estrogen-linked regulation; decline post-menopause	Mattingly et al., 2008; Singulani et al., 2020
Mitophagy/quality control	PINK1, Parkin, LC3, BNIP3	Impaired clearance of damaged mitochondria	ROS accumulation; neuroinflammation	Possible sex differences; limited direct data	Pradeepkiran and Reddy, 2020; Adlimoghaddam et al., 2025
Mitochondrial ROS & redox balance	SOD2, GPx, Catalase, Nrf2	Elevated ROS; weakened antioxidant defense	Lipid, protein, and DNA damage	Greater oxidative damage in males; estrogen protective	Tenkorang et al., 2018; Misrani et al., 2021b
Mitochondrial calcium handling	MCU, NCLX, VDAC	Ca^2+^ overload; mPTP activation	Apoptosis; synaptic failure	Potential estrogen-mediated stabilization	Sepulveda-Falla et al., 2014; Wang Y. et al., 2017
Mitochondrial–inflammatory crosstalk	NLRP3, cGAS-STING, mtDNA	mtDNA release; inflammasome activation	Chronic neuroinflammation	Stronger innate immune activation in females	Goldmann et al., 2013; Hanslik and Ulland, 2020
Mitochondrial apoptotic signaling	Cytochrome c, Bax/Bcl-2, Caspases	Increased membrane permeabilization	Neuronal apoptosis	Sex-dependent Bcl-2 regulation	Sharma V. K. et al., 2021; Wojcik et al., 2024
